# *Aedes aegypti* Larval Indices and Risk for Dengue Epidemics

**DOI:** 10.3201/eid1205.050866

**Published:** 2006-05

**Authors:** Lizet Sanchez, Veerle Vanlerberghe, Lázara Alfonso, María del Carmen Marquetti, María Guadalupe Guzman, Juan Bisset, Patrick van der Stuyft

**Affiliations:** * Tropical Medicine Institute "Pedro Kouri," Havana, Cuba;; † Institute of Tropical Medicine, Antwerp, Belgium

**Keywords:** Aedes aegypti control, larval indices, dengue, transmission risk, surveillance, Cuba, research

## Abstract

We assessed in a case-control study the test-validity of *Aedes* larval indices for the 2000 Havana outbreak. "Cases" were blocks where a dengue fever patient lived during the outbreak. "Controls" were randomly sampled blocks. Before, during, and after the epidemic, we calculated Breteau index (BI) and house index at the area, neighborhood, and block level. We constructed receiver operating characteristic (ROC) curves to determine their performance as predictors of dengue transmission. We observed a pronounced effect of the level of measurement. The BI_max_ (maximum block BI in a radius of 100 m) at 2-month intervals had an area under the ROC curve of 71%. At a cutoff of 4.0, it significantly (odds ratio 6.00, p<0.05) predicted transmission with 78% sensitivity and 63% specificity. Analysis of BI at the local level, with human-defined boundaries, could be introduced in control programs to identify neighborhoods at high risk for dengue transmission.

## Abstract 2

Entomologic indices can identify areas at high risk for disease transmission.

While a vaccine is under research, without immediate prospect for success, vector control remains the only way to prevent dengue transmission (*1**–**3*). Vector control programs are essentially based on source reduction, eliminating *Aedes aegypti* larval habitats from the domestic environment, with increasing community involvement and intersectoral action in recent decades (*4**,**5*). However, current entomologic indicators do not seem to reliably assess transmission risks, define thresholds for dengue epidemic alerts, or set targets for vector control programs (*6**,**7*). Therefore, defining new indicators for entomologic surveillance, monitoring, and evaluation are among the research priorities of the World Health Organization Special Programme for Research and Training in Tropical Diseases.

Although only adult female *Aedes* mosquitos are directly involved in dengue transmission, entomologic surveillance has been based on different larval indices (*8**,**9*). The house index (HI, percentage of houses positive for larvae) and the Breteau index (BI, number of positive containers per 100 houses) have become the most widely used indices (*6*), but their critical threshold has never been determined for dengue fever transmission (*9**,**10*). Since HI<1% or BI<5 was proposed to prevent yellow fever transmission, these values have also been applied to dengue transmission but without much evidence (*8**,**11*). The Pan American Health Organization described 3 levels of risk for dengue transmission: low (HI<0.1%), medium (HI 0.1%–5%), and high (HI>5%) (*12*), but these values need to be verified (*13*). The vector density, below which dengue transmission does not occur, continues to be a topic of much debate and conflicting empiric evidence. For example, dengue outbreaks occurred in Singapore when the national overall HI was <1% (*14*). In contrast, researchers from Fortaleza, Brazil, found that dengue outbreaks never occurred when HI was <1% (*15*). However, different geographic levels are used to calculate the indices in the various studies, and the appropriated level for entomologic indices is in itself an issue of debate (*16*). Furthermore, the appropriateness of larval indices has been questioned; recently, as an alternative, pupal indices were developed by Focks et al. (*7*) to better reflect the risk for transmission. Still, their utility for source reduction programs is controversial, and the feasibility of pupal collection in routine *Aedes* surveillance is untested (*17*).

In this study, we assessed the usefulness of larval indices for identifying high-risk areas for dengue virus transmission. We examine the influence of measurements at different geographic levels, establish a threshold for epidemic outbreaks, and discuss their utility for community-based *Aedes* control programs.

## Methods

### Context

The Cuban dengue prevention program has been hailed as among the few success stories in *Aedes* control (*18**,**19*). It was initiated in 1981, during the first dengue hemorrhagic fever epidemic in the Americas (*20*). As a result of this effort, Cuba was free of dengue from 1982 to 1996, although *Aedes* was reported again from 1992 (*21*). In 1997, dengue transmission occurred in Santiago de Cuba, a municipality located in the eastern part of the country (*22*). The epidemic remained limited to this city, but *Aedes* mosquitoes were observed in 29 other municipalities, including Havana, the capital city, in the northwest of the country. After intensification of vector control activities in the entire country (*22*), HIs from 0.05% to 0.91% were observed in Havana between 1997 and 2001 (*23*). In spite of these low indices, an outbreak of 138 cases of dengue fever occurred in September and October 2000; both dengue 3 and dengue 4 viruses were isolated (*1*). Dengue serotypes 3 and 4 had never circulated in Cuba, and we can assume low or nonexistent immunity in the population. From June 2001 to February 2002, a new outbreak occurred, and 12,889 new dengue cases were confirmed (*23*).

### Study Area

The study was conducted in Playa Municipality, in the northwest of Havana. The municipality has an area of 34.90 km^2^ and a population of 182,485 inhabitants. It has an average annual temperature of 25°C and precipitation of 132.9 mm in the rainy season (May–October). The population density is 5,228 habitants per square kilometer. The municipality has a noncontinuous water supply (every 2 days) and irregular garbage collection. It is divided into 9 health areas, each providing primary care to ≈30,000 people. We performed an in-depth study in the 5 health areas where dengue transmission occurred in the September–October 2000 epidemic.

### Study Design

We conducted a case-control study. Two units of analysis were used: blocks of houses (a block has on average 50 houses) and neighborhoods, which were defined as a block plus surrounding blocks (this definition generally results in clusters of 9 blocks with a radius of ≈100 m). These units are defined by manmade boundaries and not by ecologic determinants, per se, to usefully guide community-based control. We defined a "case" as a block (or neighborhood) of houses in the study area where >1 inhabitant was detected with confirmed dengue fever during the September–October 2000 outbreak. "Control" blocks (or neighborhoods) were randomly sampled from those in the study area where no dengue case was reported.

### Data Collection

#### Dengue Fever

Dengue cases were defined as patients with fever and >2 symptoms of dengue fever such as myalgia, arthralgia, headache, and rash, with serologic confirmation by immunoglobulin M–capture enzyme-linked immunosorbent assay (*1**,**12*) at the national reference laboratory of viral diseases in the Institute of Tropical Medicine, Havana.

During the epidemic, suspected cases were identified through the health services. Additionally, a seroepidemiologic survey was conducted in the study area at the end of October 2000; all family physicians made home visits to families under their responsibility, searching for recent denguelike illnesses. Blood samples were collected from all persons with a history of fever.

All confirmed dengue patients (passively and actively found) were interviewed by their family physician, supervised by an epidemiologist of the health area, to determine the exact date of symptom onset and places visited in the 10 preceding days. The completeness of the collected information was verified by epidemiologists of the Institute of Tropical Medicine, and if necessary, patients were revisited.

### Entomologic Information

We used entomologic surveillance data that were independently recorded by the National Vector Control Program. At 2-month intervals, vector control technicians exhaustively inspected every house in the Playa Municipality for larval stages of *Ae. aegypti*. We used data collected in 3 cycles, July–August 2000 (before the epidemic), September–October 2000 (during the epidemic), and November–December 2000 (after the epidemic). We extracted information on the number of inspected houses, positive containers (with *Ae. aegypti* pupae or larvae), and houses with >1 positive container. We eliminated 4.8% of the blocks from the study because they were not inspected in the 3 inspection cycles.

### Data Analysis

We related all data collected to geographic coordinates by a unique house block code and introduced it in MapInfo software (MapInfo Corporation, Troy, NY, USA). Case-patients were located by their address in the corresponding block. For the 3 entomologic inspection cycles, HI and BI were calculated at the block, neighborhood, and health area level. Additionally, we identified the BI_max_, which is the highest or maximum BI at the block level for each neighborhood of the case and control blocks included in the study. This variable is derived with the following equation: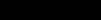,where BI*_i_* is the BI of the *i*th block belonging to the concerned neighborhood *N*, and ∀*i*⊂*N* indicates that all BI*_i_* of *N* are considered to identify the BI with the highest value as BI_max_.

All data were exported to SPSS (SPSS Inc., Chicago, IL, USA) for analysis. We calculated the Spearman rank correlation coefficient between the different indices in the 3 inspection cycles. The entomologic indices were transformed to approximately normal distributions (by using square root transformation) for calculating means, standard deviations, and 95% confidence intervals. Differences in the distribution of the indices were assessed with the Mann-Whitney test.

We assessed the discriminative power of the indices by using receiver operating characteristic (ROC) curves. Their accuracy to discriminate between case and control blocks (and neighborhoods) was classified according to the value of the area under the ROC curve (AUC) (*24*) as noninformative (AUC<0.5), less accurate (0.5<AUC<0.7), moderately accurate (0.7<AUC<0.9), highly accurate (0.9<AUC<1) and perfect (AUC = 1). The value of the indices with the highest sensitivity, >50% specificity, for discriminating case and control geographic units was taken as the optimal cutoff point. The lower limit of 50% specificity was set to safeguard positive predictive value and decrease the number of units falsely classified at high risk for dengue transmission, which triggers unnecessary action and generates unproductive costs. The association between the entomologic indices and dengue transmission was further explored by logistic regression models.

## Results

During the epidemic, health services assisted 4,679 febrile patients in the 5 health areas included in the study. All patients were serologically tested 5 days after onset of fever, and dengue infection was confirmed in 47.

In the seroepidemiologic survey, 82.5% of the families were effectively visited by their family physician. The survey found 7,008 persons with symptoms of fever between September and October 2000 who had not previously attended the health services. Serum specimens were collected from all of them, and dengue infection was confirmed in 22.

As a result, 69 (47 passively identified plus 22 actively identified) dengue cases were confirmed, all patients were interviewed, and 4 cases epidemiologically related to outbreaks in other municipalities were excluded from the study. The final sample consisted of 65 confirmed dengue fever patients who lived in 38 different blocks in the 5 health areas included in the study.

In the July to August inspection cycle, before the outbreak, the overall municipal BI and HI were 0.92 and 0.87%, respectively (Table 1). The mean values of the indices calculated at the health area level were also ≈1 for areas with or without dengue cases during the subsequent epidemic. However, the mean BI and HI were >1 for case neighborhoods and substantially <1 for neighborhoods without cases. During the epidemic, the effect of the level of measurement of the indices was still more pronounced. The HI and BI at the municipality level were 1.53% and 1.73, respectively, but all health areas with dengue cases attained a BI >1. Even more marked differences existed at the block and neighborhood levels, and after the outbreak the indices returned to average values <1 at all levels of measurement. The mean values for case blocks and neighborhoods were, in all instances, consistently substantially and significantly higher (all p<0.05) than those for corresponding control units. A high correlation was observed between block-level BI and HI values (*r*>0.94, p<0.05). In most positive houses (89.6%), only 1 container with *Aedes* larvae or pupae was found.

**Table 1:** Mean house index (HI) and Breteau index (BI) before, during, and after the dengue outbreak and mean area and population at different geographic levels, Playa Municipality, Havana, 2000

Level		July–August 2000 (before outbreak)		September–October 2000 (during outbreak)		November–December 2000 (after outbreak)			
		HI (%)	BI	HI (%)	BI	HI (%)	BI	Area (km^2^)	Population
Municipality		0.87	0.92	1.53	1.73	0.69	0.73	34.90	182,485
Health area*									
	With cases (n = 5)	0.92	0.99	1.97	2.34	0.48	0.50	2.85	21,815
	Without cases (n = 4)	1.03	1.08	0.89	1.06	0.87	0.93	5.13	16,320
Neighborhood†									
	With cases (n = 38)	1.12	1.12	4.00	4.53	0.80	0.84	0.078	2,057
	Without cases (n = 38)	0.64	0.69	1.39	1.52	0.74	0.81	0.062	1,466
Block†									
	With cases (n = 38)	0.33	0.34	2.40	2.92	0.62	0.66	0.010	271
	Without cases (n = 38)	0.13	0.20	0.35	0.42	0.32	0.33	0.008	195

*For all areas in the municipality. †For neighborhoods/blocks included in the study.

The Figure shows the spatial distribution of *Ae. aegypti* larval infestation during the inspection cycles before, during, and after the epidemic and the location of the dengue fever cases in the first (September) and second (October) month of dengue virus transmission. In most blocks (70%), no *Aedes* infestation was present before the epidemic period, but 8.8% of blocks had BI values >4, with a maximum BI of 50. Of the 17 confirmed dengue patients in September, only 3 (18%) lived in a block with BI>4 in the July–August inspection cycle. However, 15 (88%) lived in a neighborhood with at least 1 block with BI>4. The *Aedes* infestation increased during the second inspection cycle and then decreased again, concurrent with the intensified vector control activities during the epidemic. From November to December, after the outbreak, 71.6% of house blocks were *Aedes*-free, while 6.3% had BI>4.

**Figure:**
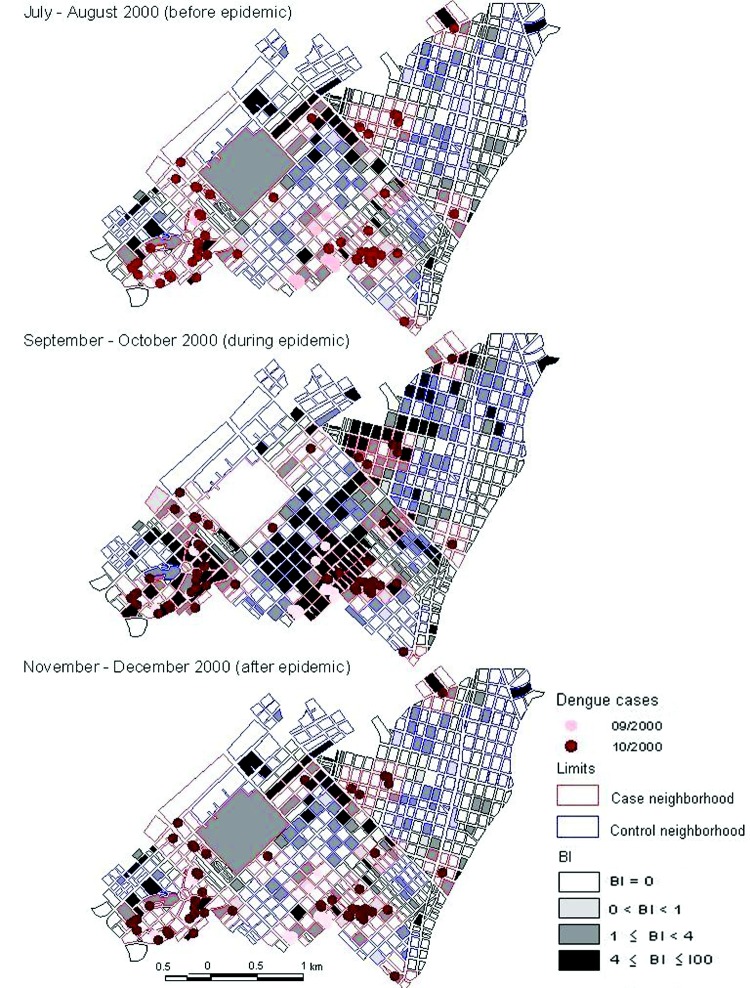
Spatial distribution of dengue cases and Breteau indices (BI) at the block level before, during, and after the dengue outbreak, Playa Municipality, Havana, 2000.

The mean block BI, the mean neighborhood BI, and the mean BI_max_ for case and control blocks are given in Table 2. Before the epidemic, the mean BI values were approximately equal for case and control units. However, the BI_max_ values were significantly higher for neighborhoods of case blocks. While transmission started in neighborhoods with high BI_max_ infestation levels, it spread into blocks and neighborhoods with lower mean BI values in October. Still, during the epidemic, the indices remained systematically and significantly higher in case blocks. After the epidemic, they returned to similar values for case and control units.

**Table 2:** Mean BI for case and control blocks before, during, after the dengue outbreak, Playa Municipality, Havana, 2000*

Block	July–August 2000 (before epidemic), mean (95% CI)			September–October 2000 (during epidemic), mean (95% CI)			November–December 2000 (after epidemic), mean (95% CI)		
	BI	NBI	BI_max_	BI	NBI	BI_max_	BI	NBI	BI_max_
September case blocks (n = 9)	0.53 (0.02–1.75)	1.52 (0.76–2.53)	6.28† (3.29–10.23)	11.95† (2.26–29.27)	10.75† (6.73–15.70)	28.4† (16.1–44.1)	0.63 (0.04–1.70)	0.64 (0.37–0.91)	2.94 (1.71–4.83)
October case blocks (n = 29)	0.29 (0.05–0.72)	1.01 (0.60–1.54)	4.24 (2.48–6.46)	1.39† (0.50–2.71)	3.16† (1.99–4.61)	12.2† (7.79–17.6)	0.66 (0.06–0.91)	0.76 (0.44–1.06)	2.87 (1.50–4.35)
Control blocks (n = 38)	0.20 (0.02–0.58)	0.69 (0.42–1.02)	2.96 (1.71–4.56)	0.42 (0.07–1.05)	1.52 (0.91–2.29)	1.52 (3.57–8.32)	0.33 (0.06–0.82)	0.68 (0.36–1.18)	2.34 (1.43–4.27)

*BI, Breteau index; CI, confidence interval; NBI, neighborhood BI; BI_max_, maximum BI at the block level for each neighborhood. †Significantly different from corresponding values for control blocks (p<0.05).

The entomologic indices from inspection cycles before and during the epidemic were less to moderately accurate at predicting subsequent transmission. The highest AUC value, 0.71, was attained with the BI_max_ from the July to August inspection cycle. At the cutoff of 4.07, it reached a sensitivity of 77.8% and a specificity of 63.2% for predicting September transmission. A neighborhood BI>1.30 gave similar results. Block-level BIs were less accurate. Comparable cutoff points for the indices in the September to October inspection cycle discriminate best for predicting transmission in October (data not shown). After the epidemic, in the November to December inspection cycle, the indices had a high specificity: 89.6% for BI<1 and 85.7% for BI_max_<4, which points toward their usefulness in nonepidemic periods.

Table 3 shows the odds ratios (OR) for dengue transmission at optimal BI cutoff values. From July to August, consistent with previous results, only BI_max_>4 was a significant predictor for identifying blocks with a case in September (OR 6.00, p<0.05). In contrast, the OR for all the different September–October BIs were significant; blocks above threshold had 3–5 times the chance of having a dengue case in October. Additionally, during the outbreak, the presence of a single positive container in a block was associated with a higher risk for dengue transmission (OR 3.49, p<0.05).

**Table 3:** OR for dengue transmission at the optimal cutoff values of the BI, Playa Municipality, Havana, 2000*

Index and cutoff value†	OR (95% CI)
July–August 2000 inspection cycle (before epidemic)	
BI per block >0	
September transmission	2.57 (0.57–11.70)
October transmission	1.69 (0.58–4.94)
BI per neighborhood >1	
September transmission	3.00 (0.66–14.17)
October transmission	1.08 (0.40–2.90)
BI_max_>4	
September transmission	6.00 (1.09–32.98)‡
October transmission	1.21 (0.45–3.25)
September–October 2000 inspection cycle (during epidemic)	
BI per block >0	
October transmission	3.49 (1.20–10.10)‡
BI per neighborhood >1	
October transmission	5.06 (1.46–17.38)‡
BI_max_>4	
October transmission	3.44 (1.23–9.63)‡

*OR, odds ratio; BI, Breteau index; CI, confidence interval; BI_max_, maximum BI at the block level for each neighborhood. †Optimal cutoff value determined as specified in Methods. ‡p<0.05.

## Discussion

We show that entomologic indices, BI in particular, allow identification of geographic units at high risk for dengue transmission. However, in regions with low *Ae. aegypti* density, identifying such units requires analysis at different levels, i.e., for blocks and neighborhoods, and short intervals between inspection cycles. Optimal cutoff values were identified for our study setting.

The existence of detailed surveillance data before, during, and after the dengue epidemic in Playa Municipality offered a unique opportunity to analyze entomologic information at different geographic levels. Entomologic data collected through routine systems, however, has some limitations. First, larval prevalence was possibly slightly underestimated: blocks were inspected by different vector control technicians, procedures used may not have been completely standardized, and few data are (randomly) missing. Second, when dengue cases were reported, the control program intensified, and more *Aedes* foci may have been detected. Third, sampling *Aedes aegypti* can be time sensitive (*25*), and our inspection cycles at 2-month intervals may not have fully captured the temporal variability of the entomologic indices. Besides, we may not have been able to identify all dengue patients who were infected outside their area of residence. Also, the study design did not allow us to detect asymptomatic dengue infections, which likely occurred in some control blocks and neighborhoods. However, we expect the potential misclassification to be nondifferential, i.e., independent of the entomologic indices. Furthermore, the experience of the technicians of the vector control program, their close supervision (including systematic revisiting of 33.3% of the inspected houses), and the interviews conducted with all dengue patients to exclude outside infection guarantee that biases, if any, are minimal.

Various researchers have investigated the relationship between dengue transmission and the *Aedes* population, expressed as larval (*15**,**26**–**31*), pupal (*7**,**13**,**32*), and adult indices (*33*). Moore (*28*) in Puerto Rico and Pontes (*15*) in Fortaleza, Brazil, used temporal graphics to compare the seasonal fluctuation of rainfall, *Aedes* larval indices, and dengue incidence. They observed a strong relation in the patterns of the 3 series. In Puerto Rico, the peak incidence of confirmed infection followed the peak larval density by ≈1 month. In Salvador, Brazil, sentinel surveillance in 30 areas detected a significant 1.4× higher seroincidence when the HI was >3% (*31*). Recently, Scott and Morrison (*16*) showed that traditional larval indices in Peru are correlated with the prevalence of human dengue infections. The variety of thresholds proposed in these and other studies could be partially explained by different methods and geographic levels of analysis used, but other factors influence the relationship between *Aedes* density and transmission risk, such as herd immunity (*11*), population density (*31*), mosquito-human interaction (*34*), virus strain, and climate, which affects mosquito biology and mosquito-virus interactions (*16*).

Entomologic indices, however, were strongly associated with transmission, and we used ROC analysis (*24*) to assess the potential of these indices to predict in which blocks transmission would occur and to select an operating point that would provide an optimum tradeoff between false-positive and false-negative results (*35*). BI_max_>4 followed by neighborhood BI≈1 during the preceding ≈2 months provides good predictive discrimination. At longer intervals, the sensitivity of these indices becomes too low. More frequent inspection cycles might perform better since *Aedes* needs only 9–12 days to develop from egg to adult (*36*). Care should, however, be taken when extrapolating these findings to communities with other herd immunity levels or different environmental conditions.

Our data also show that the geographic level of analysis determines the *Aedes* indices obtained. Marked heterogeneity is not only found inside Playa Municipality but also inside smaller health areas. Indices at the neighborhood level perform best, followed by indices at the block level. Geographic scale has too often been neglected when dengue transmission is studied. In general, overall indices are calculated for communities (sometimes of different sizes) defined by administrative boundaries, which do not constitute entomologically homogeneous units. Notwithstanding, local variability of larval indices can be inferred from the literature, in which it is sometimes mentioned. Chan et al. (*27*) noted that HI in different sections of Singapore's Chinatown varied from 10.2% to 25.0%. However, Goh et al. (*30*) reported an overall HI of 2.4% in Singapore, but at the level of 7 blocks taken together (approximately the same scale as our neighborhood), HI up to 17.9% were found. Tran et al. (*36*) defined 400 m and 40 days as the spatial and temporal boundaries of maximum dengue transmission in a dengue focus. Perez et al. (*37*) identified areas in Havana with heterogeneous risks for vector infestation by using a geographic information system. Spatial heterogeneity has also been observed at the household level for both *Aedes* populations (*10**,**38**,**39*) and dengue transmission (*26**,**29**,**40*), but this level seems less suitable for identifying areas for intervention. Blocks or neighborhoods, given the epidemiologic situation in our study area, are a more appropriate scale.

The unit of analysis used in our study, the block, is based on manmade boundaries. While these may not describe the ecology of risk, they seem to be useful markers from the perspective of community-based control interventions. In most settings, appropriately sized and locally meaningful geographic units could be similarly defined for entomologic surveillance, but the use of different boundaries or different analytical techniques could produce different results.

In our study, BI>1 and BI_max_>4 seemed to be a suitable action threshold and target, respectively, in community based dengue prevention. However, these results are derived from the analysis of 1 epidemic, and the thresholds identified may not constitute suitable targets in another epidemic or in locations where different ecologic conditions prevail. Similar studies in future epidemics and in other settings are necessary to verify the general applicability of our results.
